# Sintilimab plus Lenvatinib conversion therapy for intermediate/locally advanced hepatocellular carcinoma: A phase 2 study

**DOI:** 10.3389/fonc.2023.1115109

**Published:** 2023-02-16

**Authors:** Lijun Wang, Hongwei Wang, Yong Cui, Ming Liu, Kemin Jin, Da Xu, Kun Wang, Baocai Xing

**Affiliations:** ^1^ Key Laboratory of Carcinogenesis and Translational Research (Ministry of Education/Beijing), Department of Hepatopancreatobiliary Surgery Unit I, Peking University Cancer Hospital & Institute, Beijing, China; ^2^ Key Laboratory of Carcinogenesis and Translational Research (Ministry of Education/Beijing), Department of Radiology, Peking University Cancer Hospital & Institute, Beijing, China

**Keywords:** sintilimab, lenvatinib, hepatocellular carcinoma, conversion therapy, resection

## Abstract

**Introduction:**

Patients with intermediate or locally advanced hepatocellular carcinoma (HCC) who are not eligible for radical treatment typically have a poor overall prognosis. Treatment strategies that can convert unresectable HCC into resectable HCC may improve patient survival. We conducted a single arm phase 2 trial to evaluate the efficacy and safety of Sintilimab plus Lenvatinib as conversion therapy for HCC.

**Methods:**

A single-arm, single-center study conducted in China (NCT04042805). Adults (≥18 years) with Barcelona Clinic Liver Cancer (BCLC) Stage B or C HCC ineligible for radical surgery with no distant/lymph node metastasis received Sintilimab 200 mg IV on day 1 of a 21-day cycle plus Lenvatinib 12 mg (body weight ≥60 kg) or 8 mg (body weight <60 kg) orally once daily. Resectability was based on imaging and liver function. The primary endpoint was objective response rate (ORR), assessed using RECIST v1.1. Secondary endpoints included disease control rate (DCR), progression-free survival (PFS), event-free survival (EFS) in patients who underwent resection, surgical conversion rate, and safety.

**Results:**

Overall, 36 patients were treated between August 1, 2018, and November 25, 2021; the median age was 58 years (range, 30–79), and 86% were male. The ORR (RECIST v1.1) was 36.1% (95% CI, 20.4–51.8) and the DCR was 94.4% (95% CI, 86.9–99.9). Eleven patients underwent radical surgery and one received radiofrequency ablation and stereotactic body radiotherapy; after a median follow up of 15.9 months, all 12 were alive and four had recurrence, median EFS was not reached. Median PFS among 24 patients who did not undergo surgery was 14.3 months (95% CI, 6.3–26.5). Treatment was generally well tolerated; two patients had serious adverse events; there were no treatment-related deaths.

**Conclusions:**

Sintilimab plus Lenvatinib is safe and feasible for the conversion treatment of intermediate to locally advanced HCC initially unsuitable for surgical resection.

## Introduction

Patients with early-stage HCC can often achieve long-term survival or even a complete cure after receiving radical treatment, such as surgery or liver transplantation (1). In contrast, for patients with intermediate−advanced HCC, the median survival time is only 10–24 months (1). Unfortunately, the majority of patients with HCC in China have advanced disease at diagnosis, are not eligible for radical treatment, and typically have a poor overall prognosis (2). As a result, treatment strategies that can convert unresectable HCC into resectable HCC may improve survival compared with palliative treatment alone.

Traditionally, transarterial chemoembolization (TACE) has been the standard treatment recommended for intermediate HCC (3), and commonly used for conversion therapy. However, the objective response rate (ORR) for TACE is only around 20% (4). In addition, for some patients with intermediate HCC (Stage B on the Barcelona Clinic Liver Cancer [BCLC] staging system), TACE is not only ineffective, but may seriously reduce the liver reserve function. In this patient population, the overall surgical conversion rate following TACE is less than 20% (5).

For patients with HCC and concomitant portal vein tumor thrombus (BCLC stage C), TACE is contraindicated and systemic therapy with sorafenib or Lenvatinib are considered standard first-line treatments. However, the effectiveness of sorafenib alone in HCC is relatively low; the ORR is around 6.5%, the median overall survival (OS) is around 10 months, and the surgical conversion rate is extremely low (6). In recent years, combining immune checkpoint inhibitors with anti-angiogenic agents has improved the treatment of unresectable HCC, with an associated ORR of around 30%, progression-free survival (PFS) of 4.6–8.6 months, and OS of 20.0–22.0 months (7–10). These relatively high ORRs and survival times may offer an opportunity for conversion therapy in patients with locally advanced HCC. In this regard, the immune checkpoint inhibitor sintilimab, an anti-programmed cell death protein (PD-1) monoclonal antibody, has shown high anti-tumor activity in multiple tumor types (10). Furthermore, in the Phase 3 REFLECT study, the ORR for Lenvatinib in patients with HCC was 18.8%, which was higher than sorafenib and was the highest ORR for a tyrosine kinase inhibitor in HCC (11).

This study was conducted to evaluate the efficacy and safety of Sintilimab in combination with Lenvatinib in patients with locally advanced (BCLC Stage C) or intermediate (BCLC Stage B) HCC who were not candidates for radical surgery.

## Methods

### Study design and patients

This was a single-arm, single-center, non-randomized, open-label study consisting of a dose limiting toxicity (DLT) phase and an extension phase. Written, informed consent was provided by all patients before undergoing any study-specific procedures. The study protocol was approved by the ethics committee of Peking University Cancer Hospital and the study was conducted in accordance with the principles outlined in the Declaration of Helsinki. All patients provided written, informed consent before inclusion. The study was registered at ClinicalTrials.gov (NCT04042805).

The study included adults (≥18 years) with histologically/cytologically confirmed HCC of BCLC Stage B and up to seven criteria out, or Stage C with major portal vein or hepatic vein invasion not eligible for radical surgery and with no distant metastasis or lymph node metastasis. The staging of the tumor was determined based on imaging examination, and a diagnosis of unresectable liver cancer was defined as poor tumor location and inability to obtain complete margins (R0/R1 resection) or predicted future liver remnant/standard liver volume (FLR/SLV) <40% after resection. Other inclusion criteria included at least 1 measurable lesion according to the Response Evaluation Criteria in Solid Tumors Version 1.1 (RECIST v1.1), Eastern Co-operative Oncology Group performance status (ECOG PS) of 0 or 1, Child-Pugh score ≤7 points, and adequate organ and bone marrow function.

Exclusion criteria included histologically/cytologically confirmed fibrolamellar HCC, sarcomatoid HCC or cholangiocarcinoma, a history of hepatic encephalopathy, a history of liver transplantation, clinical symptoms of pleural effusion and ascites that required drainage, hepatitis B virus (HBV) DNA >2000 IU/ml, hepatitis C virus (HCV) RNA >10^3^ copies/ml, or positivity for hepatitis B surface antigen (HbsAg) and anti-HCV antibodies simultaneously, bleeding from esophageal or gastric varices due to portal hypertension within the past 6 months, any life-threatening bleeding event within the previous 3 months, including the need for blood transfusion therapy, surgery or topical therapy, ongoing medical therapy, involvement of both the main portal vein and the left and right branches by portal vein tumor thrombus or simultaneous involvement of the superior mesenteric vein, previous receipt of lenvatinib therapy and/or anti-PD-1/programmed cell death ligand 1 (PD-L1)/other immune checkpoint inhibitor therapy, or prior use of interventional embolization therapy and systemic chemotherapy.

### Assessment of dose limiting toxicity

The first six patients enrolled into the study were monitored for safety for 28 days after the first treatment. If a DLT (defined in the **Supplementary Methods**) was observed in two or more of the first six patients and was assessed by the study team to be due to cumulative exposure to study drug combination therapy, the dose of Lenvatinib in the combination therapy regimen was adjusted to 8 mg (body weight ≥60 kg) or 4 mg (body weight <60 kg).

### Systemic treatment

If no DLT was observed in the first six patients, then all subsequent patients received Sintilimab 200 mg by intravenous infusion on day 1 of a 21-day treatment cycle plus Lenvatinib mesylate capsules 12 mg (body weight ≥60 kg) or 8 mg (body weight <60 kg) orally once daily continuously throughout the treatment cycle. Treatment was continued until patients achieved surgical conversion, experienced disease progression, occurrence of intolerable toxicity, loss to follow-up, death, or other conditions that required discontinuation of treatment as specified in the protocol.

### Surgery

During the treatment period, resectability was evaluated based on imaging evaluations, and hematologic tests and indocyanine green (ICG) tests were performed to evaluate liver reserve function. Resectable HCC was defined as: (1) a complete/partial response (CR/PR) or stable disease (SD) with tumor shrinkage according to RECIST v1.1; (2) the tumor can be resected at R0/R1, the estimated FLV/SLV after resection is >40%, and there is sufficient blood flow into and out of the liver; (3) the liver reserve function can tolerate the corresponding range of liver resection; (4) the patient has sufficient physical condition to tolerate general anesthesia and surgery; (5) no other contraindications for hepatectomy.

Hepatectomy was performed by wedge-shaped local resection, segmental resection, hepatic lobectomy, or hemihepatic resection according to the tumor location, size and number. The standard of postoperative hepatic insufficiency was based on the International Study Group of Liver Surgery (ISGLS) standard (12); the classification of postoperative complications was based on the Clavien-Dindo classification standard (13).

### Adjuvant therapy

For patients undergoing surgical resection, the perioperative treatment cycle was based on the adjuvant treatment plan typically used for colorectal cancer (14). The total number of courses of Sintilimab combined with Lenvatinib before and after surgery should ideally not exceed 8 cycles. If preoperative treatment had exceeded 8 cycles and the patient achieved a pathologically confirmed R0/R1 resection, adjuvant therapy was not used. However, if an R2 resection was achieved, Sintilimab plus Lenvatinib was continued postoperatively until disease progression. For patients who converted to postoperative adjuvant therapy, treatment was initiated 4 to 8 weeks after surgery, or as decided by the investigator.

### Endpoints and measurements

The primary study endpoint was ORR evaluated by RECIST v1.1 according to investigators. Secondary endpoints were disease control rate (DCR) evaluated by investigators per RECIST v1.1 and modified RECIST (mRECIST), ORR evaluated by the investigators according to mRECIST, PFS (defined as the time from the first dose of study drug to the first documented disease progression or death from any cause in patients who did not convert to resection), event free survival (EFS; defined as the time from the first dose of study drug to first time of recurrence in patients who underwent resection), OS, surgical conversion rate (the proportion of patients able to undergo radical surgery following conversion therapy), and safety.

Imaging assessments were conducted every 9 weeks ( ± 7 days) from the first dose of study treatment and then every 12 weeks ( ± 7 days) after 48 weeks of treatment. Patients undergoing surgery had their first post-surgery imaging assessment within 30 days of surgery and every 12 weeks ( ± 7 days) thereafter. Details of pathologic analysis are provided in the Supplementary materials, a major pathologic response (MPR) was defined as the reduction of surviving tumors to ≤10% in size after preoperative treatment (15).

Safety assessments consisted of the monitoring and recording of AEs according to CTCAE v5.0, laboratory evaluations, vital signs, and electrocardiograms.

### Statistics

Taking the Chinese population data from the REFLECT study as a historic reference, the ORR of Lenvatinib monotherapy was estimated to be 19% (H0) (16). Assuming that the ORR of Sintilimab combined with Lenvatinib would reach 35%, taking a one-sided α of 0.1, a β of 0.2 (80% power), and a dropout rate of 10%, enrolment was set at 36 subjects.

ORR and DCR were estimated with corresponding 95% CIs using the Clopper-Pearson method, PFS, EFS, and OS were estimated using the Kaplan-Meier method. Duration of follow-up was calculated by the reverse Kaplan-Meier estimate of OS. All statistical analyses were performed using SPSS 25.0 (SPSS, Chicago, IL) software.

## Results

### Patients and treatment

Between August 1, 2018, and November 25, 2021, 39 patients were screened, of whom 36 were enrolled and treated. The data cut-off (June 30, 2022) enabled all patients in the study to be followed up for at least 6 months. Baseline demographics and clinical characteristics of the overall population are summarized in **Table 1**.

**Table 1:** Baseline patient characteristics.

Characteristic	Sintilimab + lenvatinib (n = 36)
Median age, years (range)	58 (30-79)
Sex, n (%)	
Male	31 (86)
Female	5 (14)
ECOG PS, n (%)	
0	24 (67)
1	12 (33)
BCLC Stage, n (%)	
B	17 (47)
C	19 (53)
CNLC Stage, n (%)	
Ib + IIa	12 (33)
IIb	5 (14)
IIIa	19 (53)
Serum alpha-fetoprotein level, n (%)	
<400 ng/mL	21 (58)
≥400 ng/mL	15 (42)
Child-Pugh class	
A	36 (100)
B	0 (0)
Etiology of HCC, n (%)	
HBV	33 (92)
HCV	1 (3)
Other	2 (6)
Macroscopic portal and/or hepatic vein invasion, n (%)	
None	17 (47)
Portal vein only	7 (19)
Vp2	6 (17)
Vp3	1 (3)
Hepatic vein only	3 (8)
Vv1	3 (8)
Portal and hepatic vein	9 (25)
Vp1, Vv1	2 (6)
Vp2, Vv1	3 (8)
Vp3, Vv1-2	3 (8)
Vp4, Vv1	1 (3)

BCLC, Barcelona Clinic Liver Cancer; ECOG PS, Eastern Cooperative Oncology Group Performance Status; HBV, hepatitis B virus; HCC, hepatocellular carcinoma; HCV, hepatitis C virus.

A total of 11 patients (31%) underwent surgery with curative intent, while one patient (3%) not amenable to surgical resection due to tumor location received curative treatment with radiofrequency ablation (RFA) plus SBRT. At the data cutoff, eight patients (22%) were still receiving treatment, 16 patients (44%) had discontinued first-line treatment, five patients (14%) had died, and 23 patients (64%) remained in follow-up.

The primary reasons for treatment discontinuation are listed in **Supplementary Table 1**. Following discontinuation of first-line Sintilimab plus Lenvatinib, the cancer was still confined to the liver in the majority of patients (14/16), and 10 patients (63%) received local treatments including TACE, HAIC, and SBRT. Among them, one patient underwent salvage resection and subsequently responded to HAIC plus regorafenib combined with SBRT for portal vein tumor thrombosis.

### Safety and feasibility

No DLTs were reported among the six patients enrolled into the DLT phase of the study and a further 30 patients were recruited into the expansion phase and received treatment at the initial planned dose. Safety outcomes are reported for the overall group of 36 patients pooled from both phases.

The median duration of treatment exposure in all patients was 6.7 months (range, 1.9–24.9), and was 4.4 months (range, 3.4–8.5) in the surgical group and 7.5 months (range, 1.9–24.9) in the non-surgical group. Lenvatinib dose reductions were required in 14 patients (39%); due to weight loss <60 kg in two patients and Lenvatinib treatment-related AEs in 12 patients, Sintilimab was discontinued due to an irAEs in only one patient (3%).

The vast majority of patients (n = 34, 94%) experienced at least one treatment-related AE, of which the most common were increased blood thyroid-stimulating hormone (TSH) (47%), proteinuria (42%), hypertension (42%), and decreased neutrophil count (31%) (**Table 2**). Grade 3 treatment-related AEs occurred in seven patients (19%) and most commonly included increased alanine aminotransferase (8%), increased aspartate aminotransferase (6%), and increased total bilirubin (6%). There were no treatment-related Grade 4 AEs or fatal AEs. Serious AEs were reported in two patients (6%), one of whom (3%) had a treatment-related serious AE of immune-related hepatic toxicity characterized by total bilirubin and alanine aminotransferase elevation to >5 times the baseline level.

**Table 2:** Most Common Treatment-related Adverse Events.

Preferred term, n (%)	Sintilimab + lenvatinib (n = 36)			
	Grade 1–2	Grade 3	Grade 4	Grade 5
Increased blood TSH	17 (47)	0	0	0
Proteinuria	14 (39)	1 (3)	0	0
Hypertension	14 (39)	1 (3)	0	0
Decreased neutrophil count	10 (28)	1 (3)	0	0
Decreased platelet count	7 (19)	0	0	0
Pyrexia	6 (17)	0	0	0
Asthenia	8 (22)	0	0	0
Decreased weight	9 (25)	0	0	0
Diarrhea	4(11)	0	0	0
Increased alanine aminotransferase	2 (6)	3 (8)	0	0
Increased aspartate aminotransferase	2 (6)	2 (6)	0	0
Increased lactate dehydrogenase	2 (6)	0	0	0
Increased gamma-glutamyl transferase	1 (3)	1 (3)	0	0
Increased total bilirubin	1 (3)	2 (6)	0	0
Ascites	1 (3)	0	0	0
Hyponatremia	0	1 (3)	0	0

AE, adverse event; TSH, thyroid-stimulating hormone.

### Tumor response

**Figure 1:**
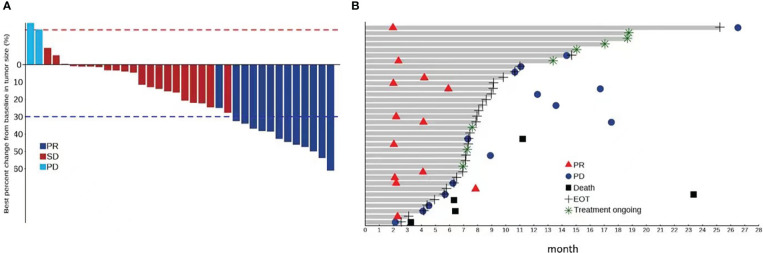
**(A)** Percentage change from baseline in sums of diameters of target lesions by RECIST v1.1 assessed by the investigators; **(B)** Swimmer plot of treatment durations, best overall response and progression of patients by RECIST v 1.1 assessed by the investigators.

According to RECIST v1.1, the ORR in all patients was 36.1% (all PRs) and the DCR was 94.4% (**Table 3**). According to mRECIST, the ORR was 66.7%, with a CR observed in eight patients (22.2%), and the DCR was 94.4%. A swimmer plot summarizing treatment duration, best overall response and time of progression is shown in **Figure 1**. Among responding patients, remission was achieved after 6 cycles in half of the patients, and after 12 cycles in 30% of patients.

**Table 3:** Summary of Efficacy Outcomes.

Parameter	RECIST v1.1	mRECIST
ORR (confirmed response), n (%)	13 (36.1)	24 (66.7)
[95% CI]	[20.4-51.8]	[31.6-41.4]
Best overall response, n (%)		
CR	0 (0)	8 (22.2)
PR	13 (36.1)	16 (44.4)
SD	21 (58.3)	10 (27.8)
PD	2 (5.6)	2 (5.6)
DCR, n (%)	34 (94.4)	34 (94.4)
[95% CI]	[86.9-99.9]	[86.9-99.9]

CI, confidence interval; CR, complete response; DCR, disease control rate; mRECIST, modified Response Evaluation Criteria in Solid Tumors; ORR, objective response rate; PD, progressive disease; PR, partial response; RECIST, Response Evaluation Criteria in Solid Tumors; SD, stable disease.

### Surgery

**Figure 2:**
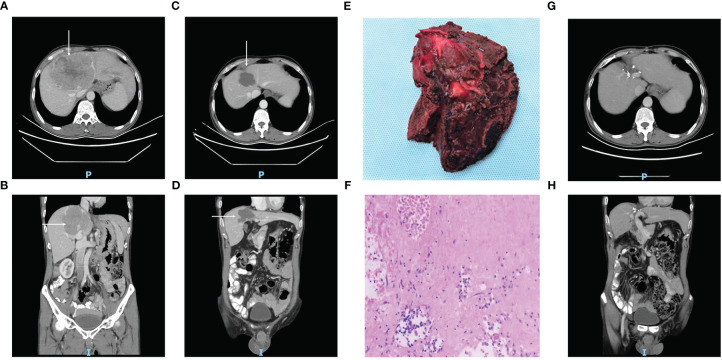
Case summary of a typical patient with unresectable HCC, who underwent surgery after conversion therapy with Sintilimab plus Lenvatinib. **(A^–D, G, H)** CT images of the axial **(A, C, G)** and coronal **(B, D, H)** planes acquired before treatment (**A**, **B**, tumor marked with white arrows), after 9 cycles of Sintilimab plus Lenvatinib (**C**, **D**, tumor shrinkage marked with white arrows), and one year after surgery (**G**, **H**, showing no signs of tumor recurrence). **(E)** Photograph of the surgical tumor specimen. **(F)** The pathology findings in the resected specimen, which indicate only 5% around tumor cell residual.

After first-line conversion therapy, resectability based on radiology was achieved in 12 patients (successful conversion rate, 33%), who underwent surgery (n = 11) or RFA plus SBRT (n = 1). Among these 12 patients, 10 were male and eight had BCLC Stage B disease at baseline, the median diameter of the largest liver nodule was 8.3 cm (range, 2.4–14.4) and the median number of lesions was 2.5 (range, 1–8). The median treatment duration required to achieve resectability was 4.4 months (range, 3.4–8.5) and the time between preoperative treatment discontinuation and surgery were 48 ± 17 days in Sintilimab and 19 ± 5 days in Lenvatinib, respectively. A typical case of a patient who underwent surgery after Sintilimab plus Lenvatinib conversion therapy is shown in **Figure 2**.

Eight patients underwent extensive liver resection (≥3 liver segments), while three patients were able to undergo only partial segmental resection as a result of tumor shrinkage. Mean (± standard deviation) blood loss was 372 ± 200 ml, and the duration of postoperative hospital stay was 8.5 ± 5 days. There were no perioperative deaths. Postoperative complications occurred in five patients, and were Clavien-Dindo Grade I in two patients (both with biliary fistulae), Grade II in two patients (both with hepatic insufficiency that improved after plasma transfusion), and Grade III in one patient (massive pleural effusion and ascites that required drainage and blood transfusion).

### Pathology

Pathologic review of resected specimens showed negative surgical margins in 11 patients. No patients had a pCR in all lesions, two patients with a single lesion had an MPR, and three patients showed significant heterogeneity of pathologic response between different lesions (i.e., pCR in some lesions, with >90% residual cancer cells in others). Resected specimens from two patients were positive for MVI, compared to four patients positive for MVI in the biopsy samples before treatment.

### Survival outcomes

The median duration of follow-up was 15.9 months (range: 3.2–34.9). As of the last follow-up, all 12 patients who underwent surgery/RFA plus SBRT were alive; four patients had recurrence, with the first recurrence limited to the liver; two of these patients had received adjuvant combination treatment as planned. Among patients without recurrence, four did not receive adjuvant chemotherapy, and four received only two adjuvant therapy cycles (eight cycles in total pre- and post-surgery). Based on RECIST v1.1, the median EFS in resected patients was not reached.

**Figure 3:**
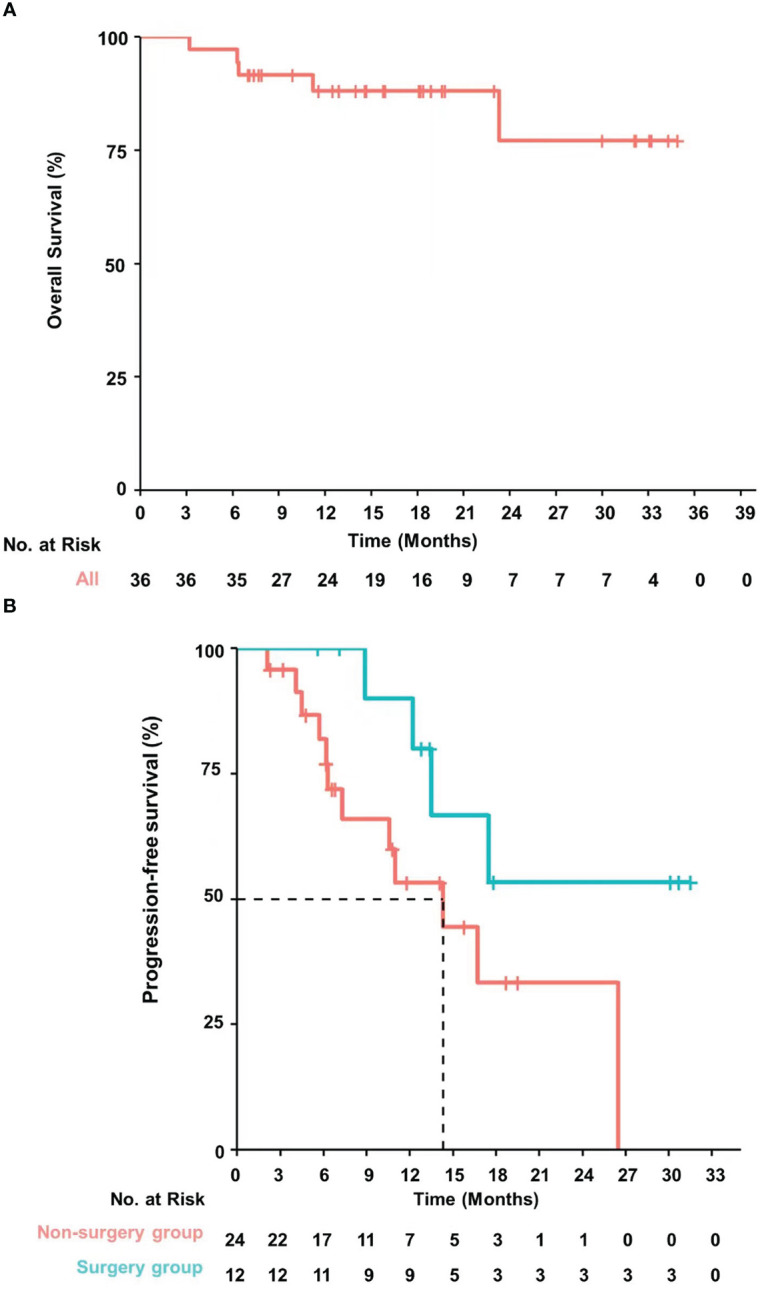
**(A)** Overall survival was not reached in the overall patient population. **(B)** Event free survival was not reached in patients who were successfully converted to surgery and median progression free survival was 14.3 (95% CI, 6.3-26.5) months in patients who failed conversion treatment.

Among all patients, the median OS was not reached. Among the 24 patients in the non-surgery group, four patients died due to tumor progression, and one due to non-tumor-related causes (cerebrovascular disease). The median PFS of the patients in the non-surgical group was 14.3 months (95% CI, 6.3–26.5) based on RECIST v1.1 (**Figure 3**). The lesions after progression in nine patients were concentrated in the liver, as shown in Supplementary materials.

## Discussion

Here, we report the first prospective study of the combination of Sintilimab plus Lenvatinib as conversion therapy for patients with unresectable HCC. Since this treatment is simpler, more accessible, and more widely applicable than local therapies, it could become a new treatment option for locally intermediate–advanced HCC.

Sintilimab plus Lenvatinib led to an ORR of 36.1% per RECIST 1.1 in patients with intermediate or locally advanced HCC, which exceeded the predefined target rate and was higher than previously reported with single-agent anti-PD-1 or Lenvatinib therapy (11). The ORR reported in the present study is also comparable to or exceeds that reported with other combinations of immune checkpoint inhibitors and anti-angiogenic drugs for the treatment of advanced liver cancer (7–9). These findings highlight the strong antitumor activity of Sintilimab plus Lenvatinib. Furthermore, the high DCR of >90% observed in the present study shows that Sintilimab plus Lenvatinib provides a clinical benefit in the majority of patients. Among responding patients, remission was achieved after 6 cycles in half of the patients, and after 12 cycles in 30%. This inter-individual variability in time to tumor regression suggests some patients may require a longer duration of treatment to achieve an optimal curative effect. However, as patients with SD have the potential for disease progression over time, there is a need to identify patients at risk of rapid progression to enable timely surgical intervention.

First-line treatment with Sintilimab plus Lenvatinib was well tolerated, with no new or unexpected AEs, in this population of patients with good liver function. In the overall study population, after adjusting the dose of Lenvatinib according to AEs and body weight changes after treatment, >90% of patients tolerated the combination, with some patients receiving treatment for >24 months. There were no treatment-related deaths and Grade 3/4 AEs were reported in only seven patients and mainly comprised proteinuria, neutropenia, and transaminase elevations, which can be identified by routine testing.

A secondary endpoint of this study was the rate of conversion to resectable HCC. Conversion therapy is referring to conversion of unresectable HCC into resectable HCC. Therefore, the definition of “unresectable” HCC is the key to the question. Prior retrospective studies have reported widely differing conversion rates, mainly due to differences in the enrolled population and their tumor burden, resulting from divergent definitions of unresectable disease (17–21). In the expert consensus (22) on the conversion therapy for HCC, the definition for “unresectable” usually contains two categories. One is surgically/technically unresectable standard that is patients cannot achieve negative surgical margin due to poor liver reserve function, insufficient remaining liver volume or other reasons. The other category is referring to oncologically/biologically unresectable standard, that is, the biological behavior of tumor is poor and better efficacy cannot be necessarily obtained after resection compared with non-surgical treatment. The latter, even with the development of the drug treatment therapy, is still controversial and has not reached a consensus. Our study included a homogeneous population comprising only patients with locally advanced–intermediate unresectable HCC. However, the criteria for unresectable HCC were technical, leading to enrollment of some patients with high tumor burden and a large number of lesions. The overall surgical conversion rate was 33% which, although not as high as in some studies, was greater than reported with TACE (4, 23) or targeted therapy alone (24), suggesting that Sintilimab plus Lenvatinib can be used for first-line conversion therapy. In addition, the patients who have successfully converted to surgery have obtained the potential for cure as well as avoid long-term drug treatment; even for patients who have failed to conversion, the above drug treatment can also prolong their survival. With the extension of follow-up time, the survival advantage of surgical patients may be more significant in future.

The optimal duration of conversion therapy remains a matter of debate. Some surgeons argue that surgery should be performed as soon as surgical criteria are met, to minimize the risk of disease progression and drug-induced liver injury (25). Others support a longer duration of treatment to pursue an optimal pathological response, based on evidence correlating the depth of pathologic remission with curative effect (15). Since we typically observed best tumor responses after 6-12 cycles, we recommend that patients undergo surgical treatment within this timeframe. To minimize the risk of tumor progression, surgery should be performed early in patients with SD or, in responding patients, when tumor shrinkage approaches a plateau. The frequent discordance of pathologic response between lesions in our study suggest that all liver tumors should be surgically removed once resectable, and that prolongation of drug therapy to deepen pathological response in the hope of improving prognosis is not warranted.

This study supports the safety of surgery after treatment with Sintilimab plus Lenvatinib. Importantly, no patients experienced tumor rupture or hemorrhage during surgery. An advantage of Lenvatinib in this setting is its short half-life, which enables its discontinuation only for >1 week before surgery, compared with 6 weeks for bevacizumab or its biosimilars. In addition, among patients in this study, preoperative Child-Pugh score remained at Grade A, and the incidence of intraoperative bleeding and postoperative liver dysfunction did not increase significantly compared with past experience of patients treated with direct surgery. On the contrary, safety may be improved by the reduced scope and difficulty of the operation afforded by tumor shrinkage. No perioperative deaths were reported in the present study. Therefore, provided adequate evaluation, surgery after conversion therapy with Sintilimab plus Lenvatinib is safe, and is not associated with greater risk.

Among the 12 patients who underwent surgery/RFA plus SBRT, post-surgical recurrence occurred in only four patients, three of whom had ≥4 lesions. Interventional chemoembolization is traditionally recommended over surgery for patients with ≥4 lesions in international guidelines (3, 22, 26). Given that recurrence rates remain high in these patients in the era of immunotherapy 1 and targeted therapy, the role of surgery requires further exploration. In contrast, for patients with 1–3 lesions, it is expected that conversion therapy followed by surgery will provide a survival benefit, but longer follow-up is needed to verify this hypothesis.

The optimal duration of adjuvant chemotherapy for HCC is not known. Due to concerns regarding cumulative toxicity, we based the duration of perioperative therapy in the present study on that of typical adjuvant treatment for colorectal cancer (i.e., 6 months) (14). In addition, a study of pembrolizumab plus Lenvatinib found that the median PFS with combination therapy was 8.6 months (7). Given that patients in our study had already received systemic treatment for nearly 6 months before surgery, it is unknown whether further, post-surgical treatment will decrease the risk of recurrence. Among patients without recurrence in our study, 4 did not receive adjuvant chemotherapy, and four patients received only two adjuvant therapy cycles. Conversely, two patients experienced recurrence during adjuvant treatment. Therefore, this study suggests eight cycles of perioperative treatment may be adequate but, given the limited sample size, more evidence is needed.

This study had several limitations. Firstly, as a single-arm, single-center, phase 2a trial, it did not include a control group against which outcomes could be directly compared. Secondly, the postoperative recurrence and survival data are not currently mature and require longer follow-up. Finally, early withdrawal of some patients and treatment delays and dropouts related to the COVID-19 pandemic could have affected the results; however, as the study design accounted for expected dropouts, we regard any such impact to be minimal.

In conclusion, Sintilimab combined with Lenvatinib is safe and feasible in the treatment of locally advanced HCC that is not suitable for surgical resection and can allow up to 33% of patients to become suitable for surgical resection. Given adequate evaluation, resection after conversion therapy is not associated with increased surgical risk, and could confer a survival benefit.

## Data availability statement

The raw data supporting the conclusions of this article will be made available by the authors, without undue reservation.

## Ethics statement

The study was approved by the ethics committee of Peking University Cancer Hospital (Approval Number: 2019YJZ29) and the study was conducted in accordance with the principles outlined in the Declaration of Helsinki. Informed consent was obtained from all individual participants included in the study. The patients/participants provided their written informed consent to participate in this study. Written informed consent was obtained from the individual(s) for the publication of any potentially identifiable images or data included in this article.

## Author contributions

LW and HW contributed equally to the work. BX contributed to the conception and design. LW and HW are responsible for the provision of the study materials and data collection. YC contributed to the imaging evaluation. ML, KJ, DX and KW provided technical and material support. LW contributed to data analysis, interpretation and draft writing. All authors read and approved the final manuscript. All authors contributed to the article and approved the submitted version.
