# Supplementary material for: Sintilimab plus Lenvatinib conversion therapy for intermediate/locally advanced hepatocellular carcinoma: A phase 2 study
Source: Front Oncol. 2023 Feb 16;13:1115109. doi: 10.3389/fonc.2023.1115109 (PMC9977802; doi:10.3389/fonc.2023.1115109)
Supplement: Supplementary file 1 [file DataSheet_1.docx]

**Supplementary methods**

***Definition of dose limiting toxicity***

A DLT was defined using the National Cancer Institute Common Terminology Criteria for Adverse Events (NCI CTCAE) version 5.0 as any of the following related to the study drug: Grade 4 hematologic toxicity lasting >7 days, Grade 3 thrombocytopenia with bleeding tendency or need for platelet transfusion, Grade 3 neutropenic fever with bacteremia or sepsis, any grade 4 immune related adverse event (irAE), Grade 3 pneumonia, Grade 2 pneumonitis that did not resolve to Grade ≤1 within 14 days after intervention, other Grade 3 irAEs that did not resolve to Grade ≤2 within 3 days after intervention or did not resolve to Grade ≤1 within 14 days (excluding asymptomatic Grade 3 thyroid or adrenal or pituitary insufficiency and Grade 3 inflammation at tumor site), other Grade 3 or 4 nonhematologic toxicity (excluding Grade 3 electrolyte abnormalities, Grade 3 or 4 infusion-related reaction, manageable Grade 3 hypertension, Grade 3 infusion site extravasation, Grade 3 arthralgia/myalgia, Grade 3 asthenia/fatigue, controlled Grade 3 vomiting, and Grade 3 or 4 elevated liver transaminases for <7 days) and any Grade 5 adverse event (AE).

***Pathologic analysis***

Pathologic analysis was conducted using the ‘7-point’ baseline method. The number and size of liver cancer lesions, the degree of differentiation of liver cancer, the extent and degree of tumor cell necrosis, and microvascular invasion (MVI) grade were routinely recorded. A pathologic complete response (pCR) was based on the complete evaluation of the histology of the resected tumor bed specimen after preoperative treatment and was defined as the absence of viable tumor cells. A major pathologic response (MPR) was defined as the reduction of surviving tumors to ≤10% in size after preoperative treatment (15).

Supplementary Table 1. Patient Disposition, Reasons for Discontinuation from

Treatment, and Second-line Treatment

| Parameter, n (%) | Sintilimab + lenvatinib (n = 36) |
| --- | --- |
| Completed surgery/RFA | 12 (33) |
| Treatment ongoing | 8 (22) |
| Primary reason for discontinuation | 16 (44) |
| Radiologic disease progression | 10 (28) |
| AE | 2 (6) |
| Withdrawal of consent | 3 (8) |
| Other | 1 (3) |
| Second-line treatment after discontinuation | 16 (44) |
| TACE ± targeted therapy | 8 (22) |
| HAIC + radiotherapy ± targeted therapy | 2 (6) |
| Other immunotherapy ± targeted therapy | 3 (8) |
| Palliative treatment | 3 (8) |

AE, adverse event; HAIC, hepatic arterial infusion chemotherapy; RFA, radiofrequency ablation; TACE, transarterial chemoembolization.
